# Skeletal Muscle Metabolomic Responses to Endurance and Resistance Training in Rats under Chronic Unpredictable Mild Stress

**DOI:** 10.3390/ijerph18041645

**Published:** 2021-02-09

**Authors:** Xiangyu Liu, Xiong Xue, Junsheng Tian, Xuemei Qin, Shi Zhou, Anping Chen, Yumei Han

**Affiliations:** 1 School of Physical Education, Shanxi University, Taiyuan 030006, China; lxy18834850353@163.com (X.L.); 15135117678@163.com (X.X.); 2 Modern Research Center for Traditional Chinese Medicine, Shanxi University, Taiyuan 030006, China; jstian@sxu.edu.cn (J.T.); qinxm@sxu.edu.cn (X.Q.); 3 The Institute for Biomedicine and Health, Shanxi University, Taiyuan 030006, China; 4 School of Health and Human Sciences, Southern Cross University, Lismore, NSW 2480, Australia; Shi.Zhou@scu.edu.au

**Keywords:** ^1^H-NMR, depression, exercise, skeletal muscle, metabolomics

## Abstract

The objectives of this study were to compare the antidepressant effects between endurance and resistance exercise for optimizing interventions and examine the metabolomic changes in different types of skeletal muscles in response to the exercise, using a rat model of chronic unpredictable mild stress (CUMS)-induced depression. There were 32 male Sprague-Dawley rats randomly divided into a control group (C) and 3 experimental groups: CUMS control (D), endurance exercise (E), and resistance exercise (R). Group E underwent 30 min treadmill running, and group R performed 8 rounds of ladder climbing, 5 sessions per week for 4 weeks. Body weight, sucrose preference, and open field tests were performed pre and post the intervention period for changes in depressant symptoms, and the gastrocnemius and soleus muscles were sampled after the intervention for metabolomic analysis using the ^1^H-NMR technique. The results showed that both types of exercise effectively improved the depression-like symptoms, and the endurance exercise appeared to have a better effect. The levels of 10 metabolites from the gastrocnemius and 13 metabolites from the soleus of group D were found to be significantly different from that of group C, and both types of exercise had a callback effect on these metabolites, indicating that a number of metabolic pathways were involved in the depression and responded to the exercise interventions.

## 1. Introduction

Depression is a common illness, with more than 264 million people affected by it globally [1]. The World Health Organization announced in January 2020 that depression is a leading cause of disability worldwide and a major contributor to the overall global burden of diseases [2]. The pathogenesis of depression has not been fully elucidated [3]. However, it has been evident that, as one aspect of its etiology, depression is closely related to metabolic disorders [4]. It has also been shown that physical exercise is a promising intervention for depression and has the advantages of lower cost, fewer side effects, and many other health benefits compared with pharmaceutical treatments [5,6,7]. At present, the research on the mechanisms of the antidepressant effects of exercise mainly focuses on the neurobiological changes [8,9,10], however, less attention is given to the potential peripheral mechanisms, such as metabolite changes in skeletal muscle.

Metabolomics is an emerging discipline that studies the overall change of metabolites in cells and the organism [11]. With metabolomics, we can explore the pattern of change in the endogenous metabolite spectrum of an organism under the influence of external environmental stimulation, gene mutation, and pathophysiological factors [12]. Skeletal muscle is a highly plastic tissue with a number of identified phenotypes and different types of exercise training, such as endurance vs. resistance training, resulting in specific responses and adaptations in the metabolic characteristics and regulatory pathways in different types of muscle fibers [13,14]. It is known that some muscles consist of predominantly slow-twitch, oxidative fibers, such as the soleus in rats, and some consist of predominantly fast-twitch, glycolytic fibers, such as the gastrocnemius, while most muscles have a mixture of fast and slow-twitch fibers [15]. Metabolites, as the end or by- products of cellular biochemical activities, have been studied to explore the relationships between genotypes and phenotypes, reflecting the regulatory effect of gene expression and protein changes on a biological system [16]. To date, most of the studies on exercise intervention for depression are on the effects of endurance training (mainly relying on aerobic energy metabolism) [17,18], although resistance training (relying on mixed anaerobic and aerobic metabolism depending on exercise intensity, duration, and repetition) has also been shown to be beneficial in the management of several health conditions, such as reducing obesity, improving sarcopenia, and so on [19,20]. It is important to explore the efficacy and understand the mechanisms of various types of exercise interventions for the prevention and treatment of depression as well as other types of health disorders. We have previously examined urine metabolomic responses to endurance and resistance training in rats under chronic unpredictable mild stress (CUMS) [21]. However, there has been no report to date that has examined the regulatory roles of skeletal muscle metabolites by using metabolomics in relation to the pathophysiological responses to exercise interventions for depression.

The objectives of this study were to compare the antidepressant effects between two types of exercise, endurance vs. resistance training, for optimizing interventions, and examine the metabolomic changes in the soleus and gastrocnemius muscles in response to the exercise, using the ^1^H-NMR metabolomics technology in combination with the multivariate statistical method, in a rat model of CUMS-induced depression. With consideration of the metabolic characteristics of different types of muscle fibers, as well as different types of exercise, it was hypothesized that the endurance and resistance exercise interventions would lead to different changes in metabolomics in the fast- and slow-twitch muscle fibers. The outcomes of the study would contribute to a better understanding of the therapeutic effects and their underlying mechanisms of exercise interventions for depression.

## 2. Materials and Methods

### 2.1. Subjects

Male Sprague-Dawley (SD) rats, SPF grade, with a body mass of 180~200 g, were obtained from Beijing Weitong Lihua Experimental Animal Technology Co., LTD (Animal License Number: SCXK, Beijing, China, 2016-0006). The rats were housed in an air-conditioned room with an ambient temperature of 23 ± 1.5 °C, humidity 45% ± 15%, and light 12 h on and 12 h off.

### 2.2. Ethical Approval

This study was approved by the Animal Experiments Ethics Committee of the University (approval number: SXULL2018006). The maximal effort was made to minimize animal suffering and the number of animals necessary for the acquisition of reliable data.

### 2.3. Chronic Unpredictable Mild Stress (CUMS) Modeling and Grouping

The method of modeling has been presented previously [21]. Briefly, 32 male SD rats were randomly divided into 4 groups, with 8 in each group: (1) Blank control group (C), (2) CUMS control group (D), (3) CUMS with endurance exercise group (E), and (4) CUMS with resistance exercise group (R). All rats in the experimental groups received CUMS modeling stress during the 1st 4 weeks. The stimuli included: No water for 24 h, fasting for 24 h, tail clipping for 2 min, 4 °C ice bath for 5 min, ultrasonic stimulation for 3 h, movement restraint for 3 h, thermal stimulation for 10 min, and electric shocks to feet (voltage 36 V, shock interval 10 s, for 10 times), day and night reversal (placed under fluorescent light for 24 h). The rats were given only one type of stimulus each day. To prevent the rats from forming memories in response to stress, the same stress stimulus was not repeated for at least 7 days.

### 2.4. Evaluation of Model Validity

The validity of the modeling was evaluated by the body weight (BW), sucrose preference test (SPT), and open field test (OFT) to assess depression-like behavior at the end of each week.

#### 2.4.1. Body Weight Measurement

The BW was measured between 3 p.m. and 5 p.m. on the last day of each week.

#### 2.4.2. Sucrose Preference Test

Anhedonia is an important symptom of depression and can be routinely measured by sugar water preference [22]. Each rat (single-caged) was placed simultaneously with 2 water bottles—the 1st 24 h with 2 bottles of 1% sucrose solution, and the 2nd 24 h with 1 bottle of 1% sucrose solution and 1 bottle of plain drinking water. At the end of the training, the subjects were deprived of water and fasted for 12 h, then subjected to a sucrose preference text by being given 2 pre-weighed solution vials: 100 mL of 1% sucrose solution and 100 mL of plain water. The 2 vials were placed at the same height. The sucrose preference rate was determined by the consumption of the 1% sucrose solution relative to the total liquid intake, measured after 3 h; i.e., sucrose preference rate (%) = sucrose intake (g)/total liquid intake (g) × 100%.

#### 2.4.3. Open Field Test

The floor of the open-field experimental device (100 cm × 100 cm) was divided into 25 equal squares. Rats were placed in the center square for 1 min to adapt to the environment. The number of crossings and standings in 5 min was recorded. A crossing referred to entering into a new square with 4 paws, reflecting the rat’s ability to exercise. The standing referred to that the forelimbs of the rat raised from the ground, reflecting their ability to explore [23].

### 2.5. Exercise Protocols

#### 2.5.1. Endurance Exercise

The endurance exercise consisted of 30 min running on a motor-driven treadmill, at zero degree slope and speed of 3 m/min for the first 5 min, 5 m/min for the next 5 min, and 8 m/min for the remaining 20 min, with a total of 30 min per day, 5 days per week for 4 weeks.

#### 2.5.2. Resistance Exercise

The resistance exercise group was given a familiarization training of 15 min a day for 3 days. They climbed a ladder of 1.1 m long, 0.18 m wide, with a 2 cm grid, and 80 degrees of inclination, 4 times on the 1st day, 6 times on the 2nd day, and 8 times on the 3rd day. In the formal training, each rat climbed 8 times a day, 5 times a week, for 4 weeks, with incremental loads. The load was added to the rat’s tail by a lead sheet wrapped in Vega tape, with a weight of 25% of the animal’s BW during the first week of training. The load was increased by 25% BW each week until 100% BW in the 4th week. The animals that stopped climbing were touched on the back by a ruler or tweezers to encourage the movement. At the top of the ladder, there was a wooden box in which the rats could rest for 2 min before the commencement of the next climbing repetition.

### 2.6. Muscle Sample Collection and Preparation

**Figure 1:**
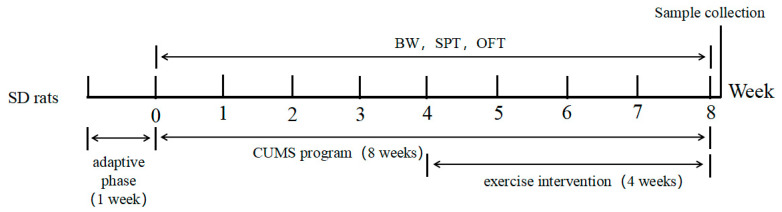
Experimental flow chart. BW, body weight; SPT, sucrose preference test; OFT, open field test.

At the end of the 4 weeks of training, the same assessments were performed for BW, STP, and OFT (Figure 1). After overnight fasting, the gastrocnemius and soleus muscle samples were obtained under deep anesthesia. The samples were cleaned from blood and trimmed for connective tissues and stored at −80 °C before metabolomics analysis.

### 2.7. NMR Metabolomics

#### 2.7.1. Sample Pretreatment

After the muscle sample was thawed, 200 mg of tissue was diced into small pieces and placed in a 5 mL Eppendorf (EP) tube, and 1 mL methanol-water (*v*/*v*, 2:1) was added. The homogenate was extracted twice on ice and transferred to a 2 mL centrifuge tube and centrifuged at 4 °C, 13,000 r/min, for 15 min. The supernatant was transferred to a 2 mL EP tube for drying with nitrogen blowing. After drying, phosphate buffer (pH = 7.40) was added to 750 μL, and the sample was centrifuged at 4 °C, 13,000 r/min, for 20 min. Then 600 μL of supernatant was transferred to a nuclear magnetic tube with diameter of 5 mm for testing.

#### 2.7.2. NMR Detection Conditions

Bruker 600 MHz AVANCE III NMR spectrometer (Bruker Biospin, Rheinstetten, Germany) was used to scan the samples 64 times with the Noesygppr1d pulse sequence. The parameters were set to a spectral width of 8 kHz, a mixing time of 150 ms, a relaxation delay of 320 ms, a sampling point of 64 k, and an accumulation number of 64. The pre-saturation method was used to suppress the water peak during the relaxation delay, the spectrometer bias was set at the water peak position, and the free induction decay signal was converted into a ^1^H-NMR spectrum by Fourier transformation.

### 2.8. Statistical Analysis

All data were expressed as the mean ± standard deviation. SPSS statistical software (Version 18.0, SPSS Inc., Chicago, IL, USA) was used for statistical analysis. Graph Pad Prism software (Version 7.0, La Jolla, CA, USA) was used to generate graphs. Data from the behavioral tests were first checked for normal distribution and then analyzed by 2-way ANOVA with repeated measures. One-way ANOVA was performed for differential metabolites. Principal component analysis (PCA), partial least squares-discriminant analysis (PLS-DA), and orthogonal partial least squares discriminant analysis (OPLS-DA) was performed using SIMCA-P multivariate statistical analysis software (Version 14.0, Umetrics, Umea, Sweden).

## 3. Results

### 3.1. Behavioral Changes

**Figure 2:**
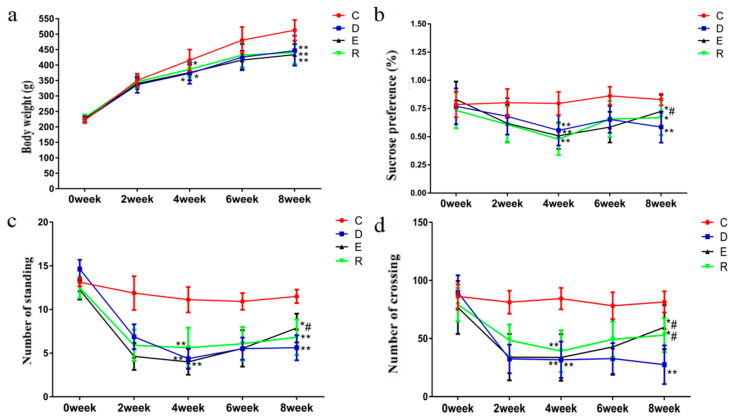
Effects of different exercise modes on behavioral indices of CUMS-induced depression rats. Comparison with group C: * *p* < 0.05, ** *p* < 0.01; comparison with group D: ^#^ *p* < 0.05, ^##^ *p* < 0.01; (**a**) body weight, (**b**) sucrose preference, (**c**) number of standing, (**d**) number of crossing.

Behavioral indicators of each group were measured once a week during the experiment. As shown in Figure 2, after the four weeks of modeling, compared with group C, the BW, sucrose preference rate, and the number of crossings and standings were significantly lower in groups D, E, and R (*p* < 0.05 or *p* < 0.01), indicating that the CUMS-induced depression model was established successfully. After the four weeks of exercise intervention period, the BW, the sucrose preference rate, and the numbers of crossing and standing of the groups D, E, and R were still significantly lower than that of group C (*p* < 0.05 or *p* < 0.01). When comparing with group D, the sucrose preference rate and the numbers of crossings and standing of group E were significantly increased (*p* < 0.05), and the number of crossings in group R was significantly increased (*p* < 0.05). (See Supplementary Materials for detailed data).

### 3.2. Skeletal Muscle Metabolomics

#### 3.2.1. Skeletal Muscle NMR Spectrum

**Figure 3:**
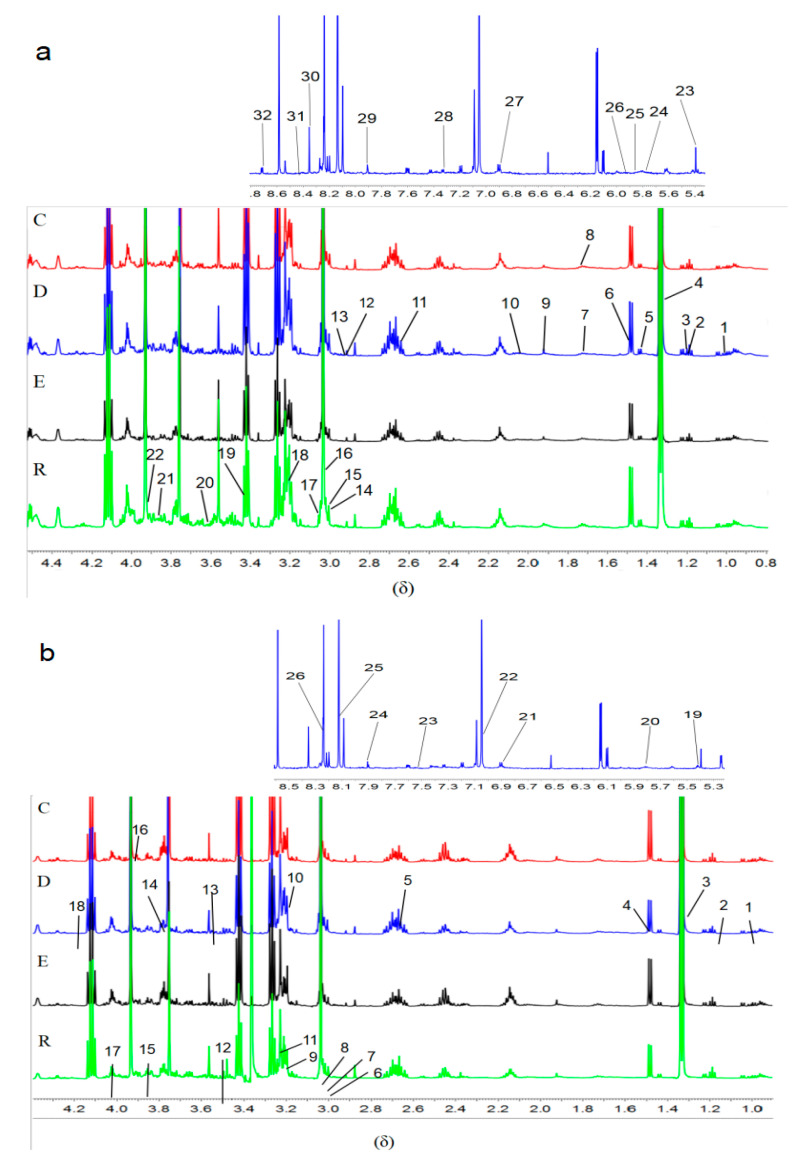
^1^H-NMR metabolite spectra (δ) of the gastrocnemius muscle (**a**) and soleus muscle (**b**). C, control group; D, CUMS control group; E, CUMS endurance exercise group; R, CUMS resistance exercise group. (**a**) (1) Isoleucine; (2) Propylene glycol; (3) very low-density lipoprotein; (4) Lactate; (5) Lysine; (6) Alanine; (7) Ornithine; (8) Acetate; (9) Ornithine acetate; (10) Glutamate; (11) Citric acid; (12) Dimethylglycine; (13) Dimethylamine; (14) γ-aminobutyric acid; (15) Anserine; (16) Creatinine; (17) Creatine; (18) Choline phosphate; (19) Taurine; (20) Threonine; (21) Alpha-glucose; (22) Betaine; (23) Allantoin; (24) Uracil; (25) Uridine; (26) Cytidine; (27) Tyrosine; (28) Phenylalanine; (29) Histidine; (30) Inosine; (31) Formate and (32) Nicotinamide. (**b**) (1) Leucine; (2) 3-Aminoisobutyrate; (3) Lactate; (4) Alanine; (5) N-acetylaspartate; (6) Aspartic acid; (7) Anserine; (8) Creatinine; (9) Choline; (10) Choline phosphate; (11) Glycerophosphate choline; (12) Glucose; (13) Glycine; (14) Glutamine; (15) Glycerol; (16) Alpha-glucose; (17) AMP; (18) Threonine; (19) Raffinose; (20) Uridine; (21) Tyrosine; (22) Histidine; (23) Hippurate; (24) Xanthine; (25) Hypoxanthine and (26) Inosine.

A typical ^1^H-NMR spectrum of skeletal muscle in CUMS-induced depression rats was shown in Figure 3. Based on HMDB (the Human Metabolome Database, http://www.hmdb.ca/ (accessed on 25 November 2020)), BMRB (Biological Magnetic Resonance Data Bank, http://www.bmrb.wisc.edu/ (accessed on 25 November 2020)), and other public databases, and consulting with related literature to identify the nuclear magnetic map of skeletal muscle, a total of 32 metabolites was identified from the gastrocnemius muscle (Figure 3a), and a total of 26 metabolites was identified from the soleus muscle (Figure 3b).

#### 3.2.2. Multivariate Statistical Analysis

**Figure 4:**
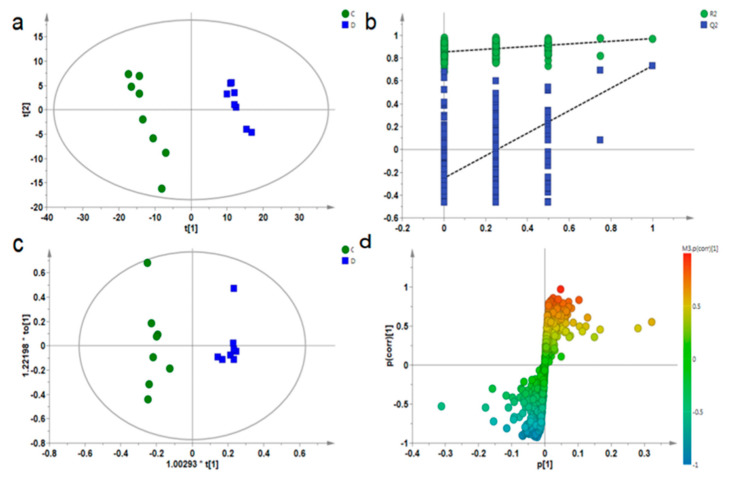
PLS-DA diagram (**a**), PLS-DA model verification diagram (**b**), OPLS-DA diagram (**c**), and corresponding S-plot diagram (**d**) for the gastrocnemius muscle samples of groups C and D.

**Figure 5:**
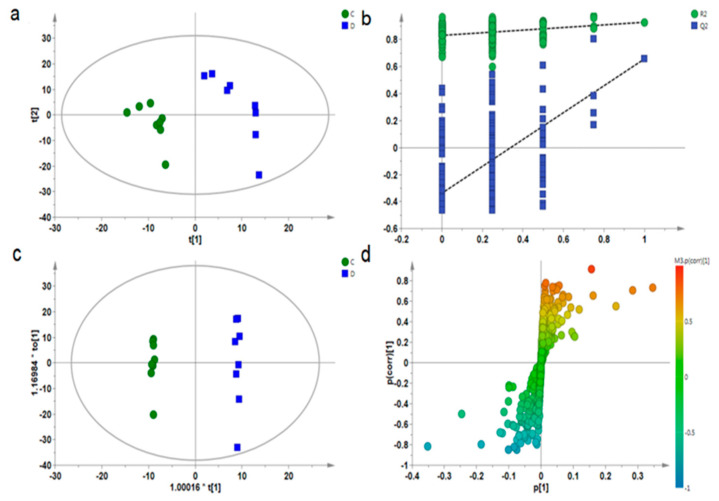
PLS-DA diagram (**a**), PLS-DA model verification diagram (**b**), OPLS-DA diagram (**c**), and corresponding S-plot diagram (**d**) for the soleus muscle samples of groups C and D.

Multivariate statistical analysis was used to find out the differences among groups, to reflect more information on the map. Partial least square discriminant analysis (PLS-DA, Figure 4a and Figure 5a) and orthogonal partial least square discriminant analysis (OPLS-DA, Figure 4c and Figure 5c) were performed on muscle samples. It can be seen from Figure 4a and Figure 5a that groups C and D were obviously separated, and R2 and Q2 generated by the Y variable were both smaller than the primary values (Figure 4b and Figure 5b), proving that the model was effective and reliable. Then, OPLS-DA analysis was performed on groups C and D, VIP values were combined with S-plot (Figure 4d and Figure 5d). According to VIP > 1 and *p* < 0.05, a total of 10 metabolites with significant differences were detected in the gastrocnemius of depressed rats, and a total of 13 metabolites with significant differences was detected in the soleus. Then, the differential metabolites were analyzed by one-way ANOVA.

As shown in Table 1 and Table 2, the levels of propylene glycol, lactate, alanine, and alpha-glucose in the gastrocnemius of group D were significantly increased compared to group C, while the levels of citric acid, lysine, creatinine, anserine, taurine, and choline phosphate were significantly reduced. After the endurance exercise intervention, five metabolites were significantly changed: the levels of propylene glycol, lactate, and alpha-glucose were decreased, and the levels of anserine and choline phosphate were increased. After the resistance exercise intervention, three metabolites were significantly changed: the level of propylene glycol was decreased, and the levels of lysine and citric acid were increased.

Compared with group C, the levels of alanine, creatinine, hypoxanthine, and glycine in the soleus muscle of group D were significantly increased, and the levels of lactate, choline, anserine, glycerophosphate choline, choline phosphate, inosine, glutamine, AMP, and histidine were significantly reduced. After the endurance exercise intervention, eight metabolites were significantly recalled: the levels of alanine and creatinine were decreased, while the levels of lactate, anserine, choline, choline phosphate, histidine, and inosine were increased. After the resistance exercise intervention, five metabolites were significantly changed: the levels of alanine, creatinine, glycine and histidine were decreased while the level of lactate was increased.

#### 3.2.3. Pathway Analysis

**Figure 6:**
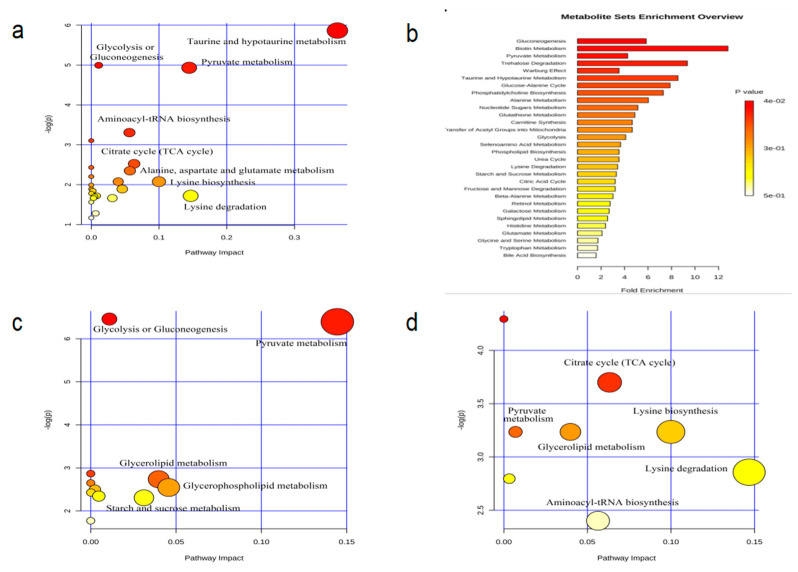
MetPA analysis of metabolic pathway ((**a**) C vs. D; (**c**) D vs. E; (**d**) D vs. R) and enrichment of metabolite sets in rat gastrocnemius muscle (**b**).

**Figure 7:**
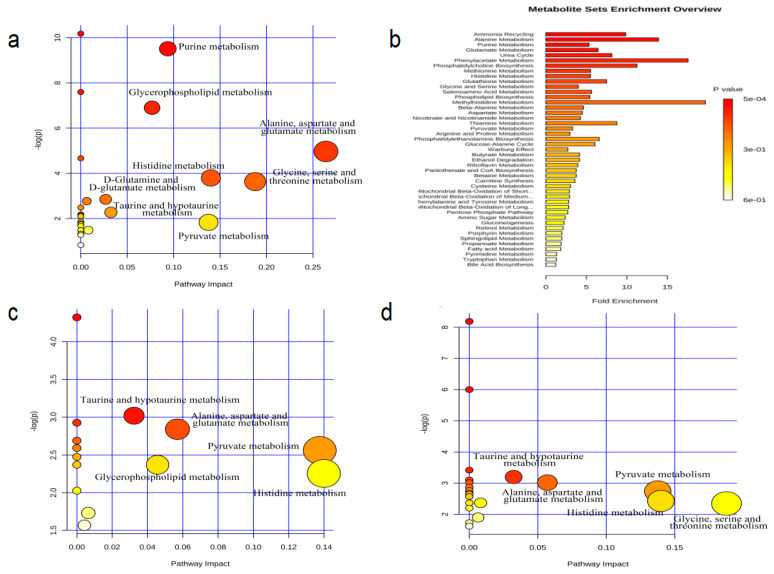
MetPA analysis of metabolic pathway ((**a**) C vs. D; (**c**) D vs. E; (**d**) D vs. R) and enrichment of metabolite sets in rat soleus muscle (**b**).

The metabolites were imported into the MetaboAnalyst 4.0 (http://www.metaboanalyst.ca (accessed on 25 November 2020)) for metabolic pathway enrichment analysis (Figure 6b and Figure 7b). On the horizontal axis, the larger the fold number, the more metabolites participated in the metabolic pathways. On the vertical axis, the darker (redder) the color, the more obvious the changes in metabolic pathways [24]. Therefore, MetPA analysis can explain the extent to which the metabolites or metabolic pathways have an impact on body metabolism. Moreover, MetPA analysis results (Figure 6a,c and Figure 7a,c) were consistent with enrichment analysis results. Holm *p*-value, FDR (False Discovery Rate), and Impact value were integrated to find that rats in group D regulated gastrocnemius muscle metabolism mainly through eight metabolic pathways (Figure 6a), including that of the glycolysis or gluconeogenesis, pyruvate metabolism, citrate acid (TCA) cycle, aminoacyl-tRNA biosynthesis, alanine-aspartate-glutamate metabolism, lysine biosynthesis, lysine degradation, and taurine-hypotaurine metabolism. Changes were observed in metabolite levels of various metabolic pathways such as glycolysis or gluconeogenesis, pyruvate metabolism, glycerophospholipid metabolism, glycerolipid metabolism, and starch-sucrose metabolism, in the gastrocnemius after endurance exercise (Figure 6c). Resistance exercise mainly improved the stress by regulating the metabolic pathways of pyruvate metabolism, TCA cycle, glycerolipid metabolism, aminoacyl-TRNA biosynthesis, lysine biosynthesis, and lysine degradation, in the gastrocnemius (Figure 6d).

The results from group D showed that there were also eight pathways were involved in the regulation of metabolism in the soleus, including that of the pyruvate metabolism, alanine-aspartate-glutamate metabolism, glycerophospholipid metabolism, glycine-serine-threonine metabolism, histidine metabolism, purine metabolism, D-glutamine-D-glutamate metabolism, and taurine and hypotaurine metabolism (Figure 7a). The improved response to stress by endurance exercise appeared to be mainly associated with the changes in the metabolic pathways of the pyruvate metabolism, alanine-aspartate-glutamate metabolism, glycerophospholipid metabolism, histidine metabolism, taurine and hypotaurine metabolism, in the soleus muscle (Figure 7c). The resistance exercise was shown to have mainly affected the metabolic pathways of pyruvate metabolism, alanine-aspartate-glutamate metabolism, glycine-serine-threonine metabolism, histidine metabolism, and taurine and hypotaurine metabolism, in the soleus, that appeared to be associated with the improvement in the symptoms of depression.

## 4. Discussion

The results of this study showed that both the endurance (group E) and resistance (group R) exercise interventions had affected the responses to stress as compared with the untreated rats with depression-like symptoms (group D) (Figure 2). Three behavioral indicators (the sucrose preference rate and the numbers of crossings and standing) in group E were significantly higher than those in group D. However, only the number of crossing in group R was significantly higher than that in group D. These results may indicate that the exercise interventions can effectively alleviate the stress in rats, and the endurance exercise appears to have broader effects than the resistance exercise.

### 4.1. Effects of Endurance and Resistance Exercise on Differential Metabolites in the Gastrocnemius Muscle

The gastrocnemius muscle mainly consists of fast-twitch fibers, which are physiologically characterized by higher contraction speed, muscle tension, fatiguability, enzyme activities of anaerobic metabolism [25], and larger glycogen reserves [26], as compared with slow-twitch muscle fibers. The significant changes of several metabolites in the gastrocnemius are worthy of further comments.

Lactate is a by-product of anaerobic glycolysis. It was found that the level of lactate in group D was higher than that in group C, indicating that the anaerobic glycolysis in the gastrocnemius of the rats under CUMS was increased, or the ability of muscle cells to clear lactate was decreased. Creatinine is a metabolite of creatine phosphate and an important energy storage substance in skeletal muscle. The decreased creatinine level in group D suggests that there may be abnormalities in the creatine phosphate energy supply system in the gastrocnemius of the rats under CUMS. Similarly, energy metabolism disorders have been found to be common in animal models of depression [27]. In our study, the four weeks of endurance exercise significantly reversed the CUMS-induced change in lactate in the gastrocnemius (Table 1), suggesting that the improved responses to stress by the endurance exercise could be related to the changes in glycolysis.

Citric acid as an antioxidant can effectively reduce oxidative stress in the body and promote metabolism through the TCA cycle [28]. At the same time, it is related to the secretion of various hormones associated with depression, such as corticotropic hormone, epinephrine, and norepinephrine [29]. Therefore, citrate may be a key mood “regulator” [30]. In this study, the CUMS resulted in a significant decrease in the level of citric acid and an abnormal increase in alpha-glucose and alanine in the gastrocnemius muscle, indicating impaired TCA cycling metabolism in muscle cells. After the four weeks of exercise, the citric acid level was significantly increased in the R group (Table 1), suggesting that resistance exercise could have improved the TCA cycle in the gastrocnemius muscle.

Taurine has a protective effect on lipid peroxidation of the cell membrane [31]. Lack of lysine and taurine in the muscle may lead to tissue damage and inflammation [32], and inflammation was one of the important factors causing depression. Compared with group D of this study, the level of lysine in the gastrocnemius of the R group was significantly increased. Although the taurine level did not change significantly, it showed a trend of callback (Table 1). It can be suggested that the resistance exercise played an antidepressant role by regulating lysine metabolism. However, there were no significant changes in lysine and taurine levels in the gastrocnemius muscle of group E, suggesting the specific effect of the resistance exercise on this pathway.

It has been reported that propylene glycol is closely associated with early central nervous system disorders in humans, such as depression, epilepsy, and other diseases [33]. Recently, there has been a report on the toxicity of propylene glycol in young and adult human bodies, which can induce apoptosis in the developing central nervous system [34]. In this study, propylene glycol in the gastrocnemius of group D was significantly higher than that of group C (Table 1). It can be speculated that the propylene glycol from skeletal muscle may exert toxic effects on the central nervous system, although this needs to be confirmed by further investigation. Compared with group D, groups E and R showed significant callback of propylene glycol, suggesting that exercise can alleviate the weakly toxic effect of propylene glycol in skeletal muscle of the rats with CUMS, and play a positive role in improving responses to stress.

Anserine is an endogenous dipeptide composed of histidine (or the methylated form of histidine) and alanine, which is found in the skeletal muscle of vertebrates [35]. Anserine and its analogs (carnosine and homocarnosine) are small molecules and water-soluble. Complement therapy with anserine and carnosine has been reported to be effective in improving cognitive impairment in the elderly [36]. Our results showed that the anserine in the gastrocnemius muscle of the rats under CUMS was significantly decreased, while it was significantly increased after four weeks of endurance exercise, suggesting that endurance exercise could improve stress by regulating the histidine metabolism pathway.

### 4.2. Effects of Endurance and Resistance Exercise on Differential Metabolites in the Soleus Muscle

It is known that the soleus, as a typical slow-twitch muscle, has different metabolic characteristics compared to the fast-twitch gastrocnemius [37]. However, the effects of exercise interventions on depression in relation to the metabolic characteristics of different types of muscle fibers have not been reported previously. Some of the specific metabolomic changes in the soleus in response to the exercise interventions are highlighted below.

Glutamate is an excitatory neurotransmitter, which has been proved to be closely related to the incidence of depression [38]. Glutamate plays an important role in the synthesis of glutamine, and the phosphorylated glutamine enzyme can hydrolyze glutamine to glutamate. The reduction of glutamine found in the soleus muscle of group D might be due to its conversion to more glutamate, however, this is contrary to the results of another study on the cerebrospinal fluid [39]. So, the specific mechanism needs to be further investigated. Pyruvate and glutamate can be catalyzed by alanine aminotransferase (ALT) to form alanine and 2-oxoglutarate [40], which plays an important role in gluconeogenesis and amino acid metabolism. Therefore, an increase in glutamate may lead to an increase in alanine content. In this study, the level of glutamate did not change significantly, while the level of glutamine was significantly decreased and the level of alanine was significantly increased in group D. The levels of alanine in soleus muscle of rats in groups E and R were significantly decreased compared with those in group D, suggesting that exercise might improve stress by regulating alanine-aspartate-glutamate metabolic pathways.

A recent study showed that exercise-induced endogenous lactate crossed the blood–brain barrier and increased PGC-1 and FNDC5 levels by activating SIRT1, which in turn enhanced BDNF signaling in the hippocampus, thereby facilitating learning and memory [41]. We found that the level of lactate in the soleus muscle of the rats under CUMS was significantly reduced and that in the groups E and R were significantly higher than that in group D, suggesting that exercise-induced improvement might be related to this pathway that might have affected the expression of BDNF in the hippocampus. Interestingly, the changes of lactate in the gastrocnemius and soleus were not the same, therefore, the above speculation would require further study.

Glycine is an amino acid that can be used as a substrate in gluconeogenesis and an important energy storage substance in the body. It has the effect of improving metabolic disorders [42] and is involved in the regulation of the inflammatory response in the recovery process after muscle injury [43]. The results of this study showed that the glycine level in the soleus muscle of group D was significantly higher than that in group C, and that in group R was significantly lower compared to group D (Table 2). It can be speculated that there was an inflammatory response in the soleus muscle under CUMS, and glycine was increased as an anti-inflammatory substance to improve the body’s response to stress. However, it has also been reported that glycine level was decreased in depression, and glycine might not play a protective role in tissues when some cells were dying [44]. Therefore, further studies are required to examine the relationship between muscle glycine level and depression.

It has been reported that people with major depression show significantly lower levels of polyunsaturated fatty acids and inosine, and polyunsaturated fatty acids may play a role in the pathophysiology of depression by regulating the purine metabolic pathway [45]. Supplementation of hypoxanthine could repair the dysfunction of cells and improve the pathological state of the body [46]. Compared with group C, hypoxanthine in the soleus muscle of group D was significantly higher, while inosine was significantly lower (Table 2). The endurance exercise appeared to be able to increase the level of inosine in the soleus significantly.

Glycerophosphate choline can cross the blood–brain barrier and provide essential choline for the synthesis of phosphorylcholine and acetylcholine. It plays a neuroprotective role through anti-inflammatory effects [47], which is conducive to the improvement of learning, memory, and cognitive functions [48]. Choline is an important component of biofilms, which can regulate cell apoptosis and promote fat metabolism. Moreover, choline is a precursor of acetylcholine, which is an important neurotransmitter in the central cholinergic system and is closely related to learning and cognitive activities [49]. It has been reported that the plasma choline level decreased in patients with depression [50]. Our results showed that the contents of glycerophosphate choline, choline phosphate, and choline in the soleus muscle of the rats under CUMS were significantly decreased, suggesting a disorder of intracellular glycerophosphate choline metabolism. In response to the endurance exercise, both choline phosphate and choline in group E showed a significant callback, indicating that the improved response to the stress might be related to the changes in the choline metabolism.

In summary, we identified for the first time significant changes of 10 differential metabolites in the gastrocnemius muscle of the rats under CUMS. These metabolites were mainly involved in the TCA cycle, glucose metabolism, and phosphocreatine metabolism. The endurance and resistance exercise as prescribed in this study could recall the changes in five and three different metabolites, respectively (Table 1). We also identified 13 differential metabolites in the soleus muscle, which were mainly involved in lipid and choline metabolism. The endurance and resistance exercise could recall the changes in seven and five different metabolites, respectively (Table 2). Overall, the endurance exercise appeared to have more extensive effects on metabolites in both gastrocnemius and soleus.

The limitations of the study may include that, first, due to the constraints of time and resources, we could only deliver four weeks of exercise intervention. Whether a longer term of CUMS and exercise intervention would result in different anti-depression effects is unknown. However, it appears that it would require a minimum of four weeks of exercise training to obtain statistically significant changes in the behavioral indices, as no significant changes were found in the first three weeks of exercise intervention (Figure 2). Secondly, only one level of exercise intensity and volume was used for either the endurance or resistance exercise, therefore, the dose-response relationship for the intervention effects could not be established in this study. Thirdly, although the soleus and gastrocnemius are typical slow oxidative and fast glycolytic muscles in rodents, the effects of exercise interventions on other muscles (e.g., with mixed slow and fast fibers) cannot be predicted from the current results.

## 5. Conclusions

The outcomes of this study indicated that both the endurance and resistance exercise as prescribed could improve behavioral indices of depression in rats under CUMS, with the endurance exercise appeared to have a better effect. The metabolomic analysis identified significant changes in 10 metabolites from the gastrocnemius and 13 metabolites from the soleus muscle in rats under CUMS compared to the blank control, suggesting an association between the metabolic profile and depression. Both types of exercise intervention had a callback effect on the changes in several metabolites (five and eight in group E and three and five in group R, in the gastrocnemius and soleus, respectively), indicating that a number of metabolic pathways in skeletal muscle were involved in the improved responses to stress, and the endurance exercise might have a broader effect.

## Figures and Tables

**Table 1:** Ten metabolites and their trends of change in the gastrocnemius muscle.

Number	Potential Biomarkers	Chemical Shift (δ)	VIP	D vs. C	E vs. D	R vs. D
1	Propylene glycol	1.25 (s)	1.08	↑ *	↓ ^##^	↓ ^#^
2	Lactate	1.33, 4.12 (d)	3.79	↑ *	↓ ^#^	↓
3	Lysine	1.45, 1.72, 1.90 (s)	1.1	↓ *	↓	↑ ^#^
4	Alanine	1.49 (d)	1.13	↑ **	↓	↓
5	Citric acid	2.67 (s)	1.29	↓ **	↑	↑ ^##^
6	Anserine	3.02, 4.52 (s)	1.3	↓ *	↑ ^##^	↑
7	Creatinine	3.05, 3.92 (s)	2.59	↓ *	↑	↑
8	Choline phosphate	3.21 (m)	1.25	↓ *	↑ ^#^	↑
9	Taurine	3.28 (s)	2.36	↓ *	↑	↑
10	Alpha-glucose	3.90, 5.25 (m)	1.3	↑ **	↓ ^##^	↓

D vs. C: * *p* < 0.05, ** *p* < 0.01; E vs. D, R vs. D: ^#^ *p* < 0.05, ^##^ *p* < 0.01; ↑: increase, ↓: decrease.

**Table 2:** Thirteen metabolites and their trends of change in the soleus muscle.

Number	Potential Biomarkers	Chemical Shift (δ)	VIP	D vs. C	E vs. D	R vs. D
1	Lactate	1.35 (d)	4.08	↓ **	↑ ^##^	↑ ^#^
2	Alanine	1.50 (d)	1.81	↑ *	↓ ^##^	↓ ^#^
3	Anserine	3.02,7.08,8.1 (s)	1.9	↓ *	↑ ^#^	↑
4	Creatinine	3.05, 3.92 (s)	4.01	↑ *	↓ ^##^	↓ ^#^
5	Choline	3.20 (t)	1.1	↓ *	↑ ^#^	↑
6	Choline phosphate	3.21 (m)	1.77	↓ *	↑ ^#^	↑
7	Glycerophosphate choline	3.23 (s)	2.74	↓ *	↑	↑
8	Glycine	3.56 (d)	1.05	↑ *	↓	↓ ^##^
9	Glutamine	3.77 (d)	3.24	↓ **	↑	↑
10	AMP	4.02,6.15,8.25 (t)	1.35	↓ **	↑	↓
11	Histidine	7.04, 7.11 (s)	1.75	↓ **	↑ ^##^	↓ ^#^
12	Hypoxanthine	8.13, 8.21 (s)	2.16	↑ **	↓	↑
13	Inosine	8.24 (d)	1.08	↓ *	↑ ^##^	↑

D vs. C: * *p* < 0.05, ** *p* < 0.01; E vs. D, R vs. D: ^#^ *p* < 0.05, ^##^ *p* < 0.01; ↑: Increase, ↓: Decrease.

## Data Availability

Data is contained within the article or supplementary material. The data presented in this study are available in [insert article or supplementary material here].

## Supplementary Materials

The following are available online at https://www.mdpi.com/1660-4601/18/4/1645/s1, Table S1: Changes in the body weight (g) of rats in different groups during the experiment, Table S2: Changes in the sucrose preference (%) of rats in different groups during the experiment, Table S3: Changes in the number of standing of rats in different groups during the experiment, Table S4: Changes in the number of crossing of rats in different groups during the experiment.
