# Supplementary material for: Skeletal Muscle Metabolomic Responses to Endurance and Resistance Training in Rats under Chronic Unpredictable Mild Stress
Source: Int J Environ Res Public Health. 2021 Feb 9;18(4):1645. doi: 10.3390/ijerph18041645 (PMC7914905; doi:10.3390/ijerph18041645)
Supplement: Supplementary file 1 [file ijerph-18-01645-s001.pdf]

Supplementary Materials

Table S1. Changes in the body weight (g) of rats in different groups during the experiment

Groups	0week	2week	4week	6week	8week
**C**	221.48±8.18	350.58±21.70	415.95±34.35	480.82±42.68	512.80±33.34
**D**	223.26±11.01	336.42±26.08	373.46±33.98*	425.68±42.35	446.61±48.52**
**E**	226.15±10.86	340.48±17.62	375.95±24.94*	416.31±29.92	433.80±33.76**
**R**	230.25±9.68	345.96±20.14	386.33±25.54*	432.36±39.35	440.67±37.68**

D vs. C: * p <0.05, ** p <0.01; E vs. D, R vs. D: # p <0.05.

Table S2. Changes in the sucrose preference (%) of rats in different groups during the experiment

Groups	0week	2week	4week	6week	8week
**C**	0.79±0.11	0.80±0.12	0.79±0.10	0.86±0.08	0.83±0.05
**D**	0.77±0.16	0.68±0.16	0.56±0.13**	0.65±0.12	0.59±0.14**
**E**	0.83±0.16	0.61±0.17	0.51±0.12**	0.59±0.14	0.73±0.15*#
**R**	0.73±0.16	0.61±0.16	0.48±0.14**	0.66±0.16	0.67±0.15*

D vs. C: * p <0.05, ** p <0.01; E vs. D, R vs. D: # p <0.05.

Table S3. Changes in the number of standing of rats in different groups during the experiment

Groups	0week	2week	4week	6week	8week
**C**	13.13±0.47	11.88±1.94	11.13±1.45	10.93±0.95	11.50±0.78
**D**	14.63±1.07	6.88±1.42	4.38±1.17**	5.52±1.27	5.63±1.44**
**E**	12.25±1.11	4.625±1.55	4.00±1.47**	5.55±2.09	7.88±1.64*#
**R**	12.50±1.21	5.90±1.78	5.63±2.28**	6.08±1.92	6.83±2.04**

D vs. C: * p <0.05, ** p <0.01; E vs. D, R vs. D: # p <0.05.

Table S4. Changes in the number of crossing of rats in different groups during the experiment

Groups	0week	2week	4week	6week	8week
**C**	86.25±10.39	81.25±9.89	84.38±9.21	78.28±11.51	81.50±9.15
**D**	89.75±14.63	32.50±12.29	31.75±15.68**	32.85±13.25	27.50±16.61**
**E**	76.75±22.74	34.00±19.95	33.75±20.05**	42.85±23.98	59.75±18.94*#
**R**	79.25±14.37	48.50±13.6	39.25±17.83**	49.35±15.22	53.00±14.65*#

D vs. C: * p <0.05, ** p <0.01; E vs. D, R vs. D: # p <0.05.
